# Darwin Versus Wallace: Esthetic Evolution and Preferential Mate Choice

**DOI:** 10.3389/fpsyg.2022.862385

**Published:** 2022-05-25

**Authors:** Adam C. Davis, Steven Arnocky

**Affiliations:** Human Evolution Laboratory, Department of Psychology, Nipissing University, North Bay, ON, Canada

**Keywords:** intersexual selection, mate choice, Lande–Kirkpatrick null model, evolutionary psychology, good genes, costly signaling

## Abstract

Dominant theorizing and research surrounding the operation of intersexual selection in evolutionary psychology tends to be guided by an adaptationist framework and aligned with models of sexual selection involving direct benefits (e.g., parental care) and indirect “good gene” and condition-dependent benefits. In this way, evolutionary psychologists more often espouse Alfred Russel Wallaces’ utilitarian viewpoint that traits become attractive because they honestly signal vigor and vitality, which gives priority to natural selection. In doing so, Darwin’s esthetic perspective originally articulated in *The Descent of Man* and alternative models of sexual selection (e.g., Fisherian runaway), are given less consideration. This is despite some informative reviews on the topic in evolutionary psychology. In the current conceptual analysis, we discuss the potential of Prum’s Lande-Kirkpatrick (LK) null model of sexual selection to help make sense of some of the mixed evidence regarding the links between attractive traits and purported markers of phenotypic and genetic condition. We then consider how the implications of the LK null model can help to shift theoretical assumptions and guide future work in evolutionary psychology on intersexual selection.

## Introduction

Both Darwin and Wallace agreed that sexual selection involves competition between same-sex conspecifics for access to reproductive opportunities (i.e., intrasexual competition; Miller, 1998; Hoquet and Levandowsky, 2015). However, they proposed competing ideas regarding the action of intersexual selection and what drives the evolution of esthetic appreciation and preferential mate choice in humans and non-human animals. Darwin believed that some traits could become attractive for nonfunctional reasons, whereas Wallace argued that traits primarily become attractive because they honestly advertise phenotypic quality, such as vigor and viability. The “Wallacean approach” has been favored in theorizing and research in evolutionary psychology for decades, but evolutionary biologists have begun to take seriously Darwin’s original stance on preferential mate choice that was first articulated in *The Descent of Man* (1871). In the current conceptual analysis, we delve into the various evolutionary processes that purportedly shape mate preferences, including Fisherian selection, sensory biases, good genes, and Zahavian handicaps. We further explore Prum’s (2010, 2012) Lande-Kirkpatrick (LK) null model of sexual selection, whereby trait–preference genetic correlations manifest without the influence of natural selection on mate preferences. We discuss the implications of the LK null model for adaptationist programs of research that predominate evolutionary psychology, whereby attractive phenotypes are often assumed to communicate underlying quality of the organism. We identify gaps in the literature on trait–preference coevolutionary dynamics and the need for more direct empirical work examining markers of health, developmental stability, and immune function in relation with salient phenotypic characteristics, such as facial features, breast morphology, and vocal register.

### Darwin and Wallace on Sexual Selection

Darwin (1871) observed that male conspecifics often battled with each other for access to females (i.e., intrasexual competition) who had the power to choose their preferred mates (i.e., intersexual selection). Darwin also documented instances of female–female rivalry, such as in some species of emu (*Dromaius*): a genus of large flightless birds. Across species, he noted that males more often possessed elaborate ornaments and competed more vigorously for access to selective female mates. In support of this idea, Bateman (1948) demonstrated how discriminating mate choice in females could produce greater reproductive variance in males and so encourage greater short-term mating effort. Trivers (1972) later provided an explanation for sex differences in sexual selection dynamics, which was based on variance in obligatory parental investment: the sex that devotes more resources to parental investment (typically females) is more discriminating in their mate choice and the less investing sex (typically males) devotes more energy to short-term reproductive effort and engages in more direct, risky, and potentially lethal intrasexual rivalry. Among the less investing sex, there is higher reproductive variability, the influence of sexual selection is stronger, and the development of conspicuous sexual characters is more apparent. This still leaves unanswered the reason(s) *why* females preferentially value certain phenotypic characteristics over others in mates. In *The Descent* Darwin proposed that females seem to possess an enigmatic “taste for the beautiful”:

Why certain bright colours should excite pleasure cannot, I presume, be explained, any more than why certain flavours and scents are agreeable; but habit has something to do with the result, for that which is at first unpleasant to our senses, ultimately becomes pleasant, and habits are inherited (p. 94).

Darwin’s contemporary, Alfred Russel Wallace (1823–1913) vacillated on the importance and operation of sexual selection. At first, he agreed with Darwin on the causes of sexual dimorphism and the vibrant coloration of plumage in male birds (Kottler, 1980). This can be seen in the following passage from Wallace (1868):

It would appear from the numerous cases in which both sexes are adorned with equally brilliant colour (while both sexes are rarely armed with equally developed offensive and defensive weapons when not required for individual safety), that the normal action of “sexual selection” is to develop colour and beauty in both sexes, by the preservation and multiplication of all varieties of colour in either sex which are pleasing to the other (p. 82).

However, from about 1876 onward, he appeared to reject sexual selection (Kottler, 1980). From this point on in his career, he adhered to a more “utilitarian” perspective and discounted the importance of females selecting males based on differential male ornamentation (Hoquet and Levandowsky, 2015). For instance, Wallace (1889) stated as:

In like manner, female birds may be charmed or excited by the fine display of plumage by the males; but there is no proof whatever that slight differences in that display have any effect in determining their choice of a partner (p. 286–287).

Wallace viewed esthetic features, such as brightly colored and prominent ornaments, as largely the products of natural selection. He believed that these traits were honest cues to vigor and vitality and were principally involved in species recognition and intimidating predators, not mate attraction. Relatedly, Wallace posited that the drab coloration of many females was not merely a default condition but served an important survival function to avoid predation (discussed in Caro, 2017). Darwin’s “esthetic” view was aligned with the notion that ornaments could become attractive for reasons that have little to do with phenotypic condition (a “taste for the beautiful”). Therefore, Darwin and Wallace expressed divergent ideas regarding the evolution of sexual dimorphism and secondary sexual characteristics (Miller, 1998; Prum, 2012; Hoquet and Levandowsky, 2015). Wallace disagreed that non-human animals could possess an “esthetic sense” and was opposed to Darwin’s position that naturalistic explanations should be used to understand human cognitive, emotional, and esthetic capacities. Unlike Darwin, Wallace argued for the power of natural selection in driving preferential mate choice and largely dismissed the influence of sexual selection (Prum, 2017). Wallace attributed various human psychological processes, such as consciousness and esthetic pleasures, to God and mystical phenomena (Fisher, 1930; Hoquet and Levandowsky, 2015).

### Adaptationism and Mate Choice

Despite more attention being given to Darwin’s ideas on the operation of sexual selection, research on preferential mate choice, particularly among evolutionary psychologists, has been notably “Wallacean.” A cursory reading of popular writing and research in evolutionary psychology on intersexual selection provides insight into how the field tends to be guided by an adaptationist bias—that mental faculties are, first-and-foremost, adaptations and the direct products of selection (Schulz, 2013). Strong adherence to adaptationism can result in ignoring or discounting the possibility that psychological traits may be exaptations instead of adaptations (Gould, 1991). Exaptations denote heritable traits that were originally selected to perform one function but have since been co-opted to perform other unrelated functions that may, nonetheless, still enhance fitness (Buss et al., 1998; Havliček et al., 2015). Psychological characteristics that promote an organism’s survival and reproduction in its current environment might also be by-products of adaptations that have no direct functional significance (i.e., a spandrel). To be sure, adaptationist thinking has, and will continue to be, essential for delineating the functional significance of purported psychological adaptations (Daly and Wilson, 1995). However, simply because a mental faculty is useful at “achieving something” that can be mapped on to indices of reproductive success should not be taken as sufficient evidence that it was shaped by selection for that specific functional purpose.

Adaptationist programs of research in evolutionary psychology tend to be aligned with the assumption that many historically and cross-culturally consistent mate preferences were selected in the ancestral environment because they provided accurate information about an individual’s phenotypic condition. Therefore, it is often assumed that: (1) particular traits become attractive because they honestly communicate “good genes,” health, reproductive value, and/or parental investment and (2) that secondary sexual characteristics correlate reliably with markers of genetic condition and phenotypic quality (Prum, 2010, 2012, 2017). In this way, evolutionary psychologists deviate from Darwin’s esthetic perspective propounded in *The Descent* and conform more to Wallace’s utilitarian viewpoint on mate choice. This bias in favor of neo-Darwinian (i.e., Wallacean) thinking likely manifests because researchers are not learning about Darwin’s original ideas, which is an important consideration for educators teaching evolutionary theory. These assumptions can be problematic. First, attractive traits may not actually qualify as adaptations and may instead be better conceptualized as exaptations or spandrels. Second, it neglects the other evolutionary processes that influence intersexual selection dynamics. In the following sections, we describe prominent models of sexual selection that tend to be favored in evolutionary psychology to explain mate preferences.

### Models of Sexual Selection

#### Good Providers, Good Genes, and Costly Signaling

Several models have been proposed to help account for preferential mate choice, specifically female choice, that vary according to the kinds of benefits that can be acquired by the selecting sex (Andersson, 1994; Jones and Ratterman, 2009). Females may acquire *direct* benefits by selecting males who are more fertile, as well as those possessing a greater capacity to invest material resources (e.g., food) and parental care (Gangestad and Thornhill, 1997; Kokko et al., 2003). Selecting males for their ability to provision resources for mates and offspring is known as the “good-provider” model of sexual selection (Hoelzer, 1989). Males may also provide direct benefits in terms of protection from predators or from other males (Frederick and Haselton, 2007). Health status may also indicate an enhanced capacity to compete for and provide ongoing resources and parental investment, and so could be desired as a good-provider indicator (Tybur and Gangestad, 2011).

Other models of sexual selection involve conferring potential *indirect* benefits to offspring *via* genetic inheritance. The model of indirect benefits commonly adhered to by evolutionary psychologists is the *good genes model*, whereby females prefer males possessing heritable traits associated with genetic quality that can be transmitted to offspring to enhance their reproductive success (reviewed in Gangestad and Thornhill, 1997). A salient issue debated by evolutionary scientists regarding the good genes model is that directional selection would presumably eliminate genetic variance for viability (i.e., the lek paradox; Kirkpatrick, 1982). However, it is clear that there exists considerable genetic variance in display traits that are under the influence of sexual selection (Prokop et al., 2012). Some have argued that heritable viability could potentially be maintained *via* mutations with the introduction of new genetic variants (Rice, 1988). Although this idea is rarely directly tested by researchers using the good genes model. Another potential issue with the good genes model is that as the selective environment changes over time so too would the viability indicators (Gangestad and Thornhill, 1997). The ongoing evolutionary arms race between pathogens and their hosts helped to delineate how markers of viability could change alongside selective pressures in the environment, and provided another mechanism through which genetic variability might be maintained (Anderson and May, 1982). Individuals selecting mates with cues to pathogen resistance could transmit better pathogenic immunity to their offspring (Hamilton and Zuk, 1982). This “parasite model” is the dominant model of good genes sexual selection adhered to in evolutionary psychology (e.g., Pazhoohi and Kingstone, 2020).

Despite its popularity in evolutionary psychology as the driving force of intersexual selection, previous meta-analytic work provided equivocal support for the role of good genes across species (Prokop et al., 2012). More recently, Achorn and Rosenthal (2020, p. 216) have argued that “For conspicuous display traits, weak signals of good genes should be the rule”. These scholars propose that when the genetic influences on viability indicators are strong, the likelihood of preferences for good genes being maintained decreases because it diminishes genetic variation *via* sexual selection. Achorn and Rosenthal (2020) take the position that the good genes model is inadequate in explaining the evolution of elaborate sexual display traits and preferences for those ornaments.

*Fluctuating asymmetry* (i.e., subtle deviations from bilateral symmetry) in physical characteristics has garnered much attention from evolutionary psychologists as a viability marker that is purportedly shaped by exposure to parasites, pathogens, toxins, and deleterious mutations (Møller, 1990). Morphological traits with lower fluctuating asymmetry are posited to be attractive because they reflect *developmental stability*—A heightened capacity to withstand genetic and environment perturbations during development (Møller, 1990; Møller and Thornhill, 1997). Individuals with higher fluctuating asymmetry have been found to suffer lower fecundity and greater mortality (Watson and Thornhill, 1994), and males with low fluctuating asymmetry, including human men, appear to benefit from greater mating success (reviewer in Gangestad and Thornhill, 1997). It is important to mention that a previous review of 40 published meta-analyses on fluctuating asymmetry in evolutionary biology indicated that about 20% of the research findings could be attributed to publication bias and that effect sizes across studies were very small (Jennions and Møller, 2002). Indeed, evolutionary researchers often overstate the importance of asymmetry in determining the attractiveness of human traits (Van Dongen, 2011). There is an evident shortage of research on sexually dimorphic secondary sexual characteristics involved in mate choice, such as the female breast, and whether fluctuating asymmetry in these characters are actually tied to phenotypic condition (Locke and Arnocky, 2021).

Zahavi (1975) argued that genetic variability in fitness could be maintained through attractive male secondary sexual characteristics that purportedly reduce survivability. Characteristics constituting *Zahavian handicaps* involve extravagant traits that are “wasteful” or produce a cost to the organism in terms of reduced survivability, and so may be honest cues to an organism’s genetic and phenotypic condition (discussed in Penn and Számadó, 2020). There are, however, some salient issues associated with the handicap hypothesis. It may be assumed that viability in males will consistently correspond to greater health (discussed in Frederick et al., 2013). However, investing in putatively costly traits can decrease an individual’s health (Kokko et al., 2002). Therefore, any evidence for a positive, negative, or neutral relation between a trait and health could be taken as evidence in favor of the handicap hypothesis. The handicap hypothesis also assumes that mate preferences are adaptive and enhance offspring viability, and evidence indicates that non-adaptive female preferences emerge under various conditions (e.g., when the development of the handicap is caused by non-heritable factors; Kirkpatrick, 1986). Furthermore, robust indicators of health and immunity are unlikely to diversify once evolved, which runs in contrast to the diversity of ornaments predicted *via* Darwin’s theory of sexual selection (Prum, 1997, 2012). The hypothesis also comes in a variety of different versions, but none seem capable of adequately explaining male sexually dimorphic ornaments (Számadó and Penn, 2018). The handicap hypothesis also tends to carry the assumption that extravagant ornaments can only be produced by genetically fit males and that these traits carry some cost to survival.

The peacock’s (*Pavo cristatus*) vibrant train was used by Zahavi (1975) to explicate the hypothesis, and it is a classic example used in evolutionary psychology as evidence of the power of sexual selection to produce complex and costly ornaments. Indeed, peacocks with a greater number of train-feather eyespots (i.e., ocelli) appear to have greater mating success (Petrie and Halliday, 1994), they help to produce larger offspring with greater survivability (Petrie, 1994), and they enjoy better health status (Loyau et al., 2005). In a study by Møller and Petrie (2002), train length, but not the number or size of the eyespots, was positively linked to body condition (body mass) and some markers of immune function (heterophil–lymphocyte ratio) but negatively related to others (humoral immunity). Other studies also indicate that there is insufficient variability in train-feather eyespot number among feral peacocks to account for variance in male mating success (Dakin and Montgomerie, 2011). The long, elaborate peacock train also does not appear to reduce locomotor performance (Askew, 2014). And because of the dichromatic nature of the visual systems of most mammalian predators that hunt birds, the peacocks colorful feathers are actually quite inconspicuous (Kane et al., 2019). These results cast some doubt on the idea that the peacocks elaborate plumage is a signal that carries a cost to survival. It is important to consider that the peacock train may not be a single ornament, but a trait that carries multiple independent signals (e.g., number of ocelli, symmetry, and vibrance of plumage; Van Doorn and Weissing, 2004). But this still does not entail that these multiple signals carry a cost to survival.

The handicap hypothesis was extended to propose that secondary sexual characteristics are honest indicators of an individual’s condition because their development is mediated by sex hormones (e.g., testosterone) that are believed to have a negative impact on the functioning of the immune system (i.e., the *immunocompetence handicap hypothesis*; Folstad and Karter, 1992). Therefore, women choosing men with well-developed sexual characters could presumably pass on these genetic and phenotypic benefits to their offspring.

The models of sexual selection reviewed so far revolve around direct benefits, good genes, and costly signaling which coincide with Wallace’s notion of utility that natural selection plays a primary role in preferential mate choice. These models are well represented in research in evolutionary psychology on the operation of mate preferences. Alternative models intended to account for female choice that resonate more with Darwin’s esthetic view have been reviewed in evolutionary psychology (e.g., Gangestad and Thornhill, 1997; Miller, 1998; Gangestad, 2001; Frederick et al., 2013), but they are given comparatively less consideration among researchers in the field.

#### Fisherian Runaway and Sensory Biases

Individuals may acquire indirect benefits for their offspring *via* mate choice that are unrelated to good genes, health, costly signals, and/or greater pathogen resistance. This was the position articulated by Darwin in *The Descent* that trait–preference covariation may result for relatively nonfunctional reasons. This model (see Gangestad and Thornhill, 1997) was elaborated upon by Fisher (1930) who argued that a female preference for a particular male ornament could become genetically correlated and co-evolve with the ornament (i.e., *Runaway selection*). This trait–preference correlation is argued to manifest because of linkage disequilibrium (i.e., non-random association of different neighboring alleles; Hosken and Wilson, 2019). Consequently, males possessing the conspicuous ornament would gain a mating advantage, and so daughters carrying the trait preference and sons possessing the ornament would increase in frequency in future generations. This results in a positive feedback process whereby the female preference and the male secondary sexual characteristic become exaggerated, which is halted and balanced by countervailing costs to survival associated with the exaggerated ornament (Fisher, 1915, 1930). Of note, coevolution is not restricted to Fisherian selection, and in nature a genetic trait–preference correlation could be at play with direct benefit and good genes models (Kokko et al., 2002).

Although the different models of sexual selection for indirect benefits tend to be pitted against one another, Kokko et al. (2003) argued that there are commonalities between them and that it is problematic to view them as mutually exclusive. For instance, it is possible that only males possessing markers of viability and good phenotypic condition can manage to produce more extravagant traits as a consequence of runaway selection (discussed in Frederick et al., 2013). Females may then express a preference for males embodying indicators of good genes presumably required for these extravagant characteristics. It can also be challenging to differentiate between the influence of direct and indirect benefits. For instance, physical formidability (e.g., greater musculature) in males may signal the ability to provide protection (Frederick and Haselton, 2007), a direct benefit, but male musculature is also considered to be an indicator of good genes (Gangestad et al., 2007), or females may select muscular males simply to provide a reproductive advantage to their offspring (i.e., Fisherian selection; Frederick et al., 2013). Although, it is worth mentioning that the evidence supporting male muscle mass as a trait that women find attractive as a signal to protection in long-term relationships is lacking (Fajardo et al., 2022). Instead, research indicates that muscular men tend to pursue short-term sexual strategies (Frederick and Haselton, 2007), and women do not appear to perceive muscular men as high in indicators of long-term partner mate value (e.g., “good father”; Gangestad et al., 2007).

Fisherian co-evolution provides a compelling alternative to other previously discussed models of sexual selection. However, it raises the question of why females display an initial preference for traits that are unrelated to viability. Across species, females appear to display a desire for more exaggerated and novel display traits, such as larger, brighter, and more colorful plumage, that can evoke stronger sensory stimulation (Ryan and Keddy-Hector, 1992; Miller, 1998). Females may also express pre-existing perceptual biases in non-mating contexts that become intertwined with sexual selection (discussed in Miller, 1998). Runaway selection (Fisher, 1915, 1930) may then amplify these sensory biases. For instance, *sensory trap* involves a female responding to an “out-of-context” stimulus provided by males during courtship, which mimics a signal that evolved to elicit a response for reasons unrelated to mate attraction (Christy, 1995). And *sensory exploitation* (Ryan and Keddy-Hector, 1992) describes how pre-existing sensory biases in females may be exploited by male courtship signals. Importantly, the idea that sensory biases could be driving preferential mate choice still necessitates appealing to natural selection along the causal chain (Prum, 2010). Invoking sensory biases to explain runaway selection also deviates from Fisher’s (1915, 1930) original position that any conspicuous trait associated with reproductive success that varies among members of a population can initiate the runaway process. These display traits can emerge through different kinds of stochastic processes, such as *genetic drift*—chance events causing fluctuations in the frequency of alleles across time in a population—such as *a bottleneck*—a sudden reduction in a population resulting in a genetically unrepresentative subsample—and *founder effects*—a random subsample of members from the original population splintering off to form a new isolated population (Kitchen, 2018). It is this position that Prum (2010) posits should serve as the null model of sexual selection.

### Darwin and the Lande-Kirkpatrick Null Model

Prum (2010, 2012, 2017) advances the idea that, in contrast to adaptationist thinking, which is often afforded epistemic privilege in evolutionary psychology, scientists should not assume that natural selection is the key mechanism governing mate choice dynamics. Following the logic of Fisherian runaway, Lande (1981) and Kirkpatrick (1982) proposed indirect models of intersexual selection whereby the strength of the genetic trait–preference correlation relative to the degree of genetic variation in the trait would dictate whether a population would proceed toward stable equilibrium (trait–preference correlation < trait genetic variance) or non-equilibrium states (trait–preference correlation > trait genetic variance). The latter condition represents the Fisherian runaway process, where a stronger correlation between the preference and trait increases the likelihood of runaway selection and a positive feedback loop that can produce extreme ornaments that must be halted by stabilizing selection (i.e., pushing a population toward intermediate phenotypes). When the trait–preference correlation is greater than the amount of genetic variance in a trait, stochastic evolutionary processes (e.g., genetic drift) may trigger runaway selection (Lande, 1981). As stated by Prum (2010, p. 3087), “…substantial evolutionary elaboration of trait and preferences can occur through drift away from a stable equilibrium and the evolution of a population toward a new equilibrium rather than a return to the former state”. Collectively, these dynamics constitute what Prum (2010) called the LK model, which he likened to the Hardy–Weinberg equilibrium: in the absence of other evolutionary forces, there exists variation in trait–preference genetic correlations. The LK model suggests that highly elaborate ornamental secondary sexual characteristics can evolve without the influence of natural selection on mate preferences and that we do not need to appeal to models of sensory bias to understand the initiation of Fisherian runaway. The action of natural selection on display traits is still encompassed within the LK model, but, unlike good gene and direct benefits models of sexual selection, it does not require the influence of natural selection on preferences for those traits. The LK model aligns with Darwin’s esthetic view of female choice, and unlike dominant approaches to intersexual selection in evolutionary psychology, it does not assume additional selective pressures beyond the trait–preference correlation or a positive association between viability and attractive characteristics (Prum, 2010). Prum (2012) stated that a truly Darwinian approach to intersexual selection should appeal to utilitarian good genes and phenotypic condition explanations only when the evidence does not support the LK null model and influence of pre-existing sensory biases. The practicality of this approach is further supported by meta-analytic work showing that Fisherian selection is likely a more important part of mate choice than the good genes model of sexual selection (Prokop et al., 2012).

In the following section, we document important mixed findings in the literature on mate preferences regarding secondary sexual characteristics commonly purported to be markers of genetic and phenotypic condition. Following Prum (2010, 2012, 2017), we believe that it is in these literatures that the utility of the LK null model may be most apparent for evolutionary psychologists.

### Secondary Sexual Characteristics, Health, Immunocompetence, and Viability

To date, limited empirical work has directly addressed questions of (1) whether human secondary sex characteristics reflect individual differences in genetic quality or immunocompetence, and (2) whether the development and maintenance of these traits truly entail an immunologic or energetic cost. Nevertheless, the role of secondary sexual characteristics in signaling heritable immunocompetence has often been framed in research literature and textbooks as being well established (see Scott et al., 2013 for review). Some work has supported a positive phenotypic correlation between secondary sex characteristics and specific markers of immunocompetence. For instance, Arnocky et al. (2018) found that in men, vocal masculinization was positively correlated with both self-reported health and salivary immunoglobulin-A (SIgA; a marker of mucosal immunity), which itself was positively correlated with testosterone (T). Given vocal physiology and corresponding fundamental frequency are influenced by T (Dabbs and Mallinger, 1999; Hodges-Simeon et al., 2021), it is possible that low pitch is attractive to females (Feinberg et al., 2005; Hodges-Simeon et al., 2011) because it serves as a costly signal of underlying immunocompetence (Arnocky et al., 2018). Listeners also rate men’s voices with a lower fundamental frequency as healthier (Albert et al., 2021). However, health may constitute a direct or indirect benefit (Tybur and Gangestad, 2011; Frederick et al., 2013), and these results could be taken as evidence in favor of either direct or indirect benefit models of sexual selection. Furthermore, evidence indicates that non-heritable factors play a more important role than heritable factors in shaping immune system functioning (Brodin and Davis, 2017).

Attractive facial characteristics have also been considered from an immunocompetence signaling perspective (see Arnocky et al., 2014 for review). Shackelford and Larsen (2000) found that facial asymmetry correlated with negative health markers (see also Jones et al., 2001; Borráz-León et al., 2021). Results regarding men’s facial masculinity have been more thoroughly studied but are equivocal (Scott et al., 2013). Rhodes et al. (2003) found that rated masculinity in the faces of young males correlated modestly with actual health. Boothroyd et al. (2005) did not support a link between women’s preferences for facial masculinity and preferences for apparent health, and there were ambiguous results regarding the relation between perceived masculinity and health. Similarly, Boothroyd et al. (2007) did not support a link between facial masculinity and health and showed how women and men perceived healthy and masculine faces to be associated with divergent personality characteristics (e.g., ambition, faithfulness, and parenting skill). Across three samples, Boothroyd et al. (2009) showed that women’s preferences for facial masculinity were negatively correlated with their preferences for facial symmetry and unrelated with their preferences for health and facial averageness. Using anthropometric measurements, Boothroyd et al. (2013) found that men’s facial masculinity predicted better past health, but worse reported health over a ten-week follow-up period. Another study examining other-rated facial sexual dimorphism found that facial masculinity was related to semen quality, but not with a salivary immune response to *Escherichia coli* or with salivary lysozyme response to *Micrococcus lysodekticus* (Foo et al., 2017). Thornhill and Gangestad (2006) found men with masculinized faces experienced fewer respiratory illnesses and less use of antibiotics. However, facial masculinization appears unrelated to heterozygosity of the major histocompatibility complex (MHC); a series of genes whereby heterozygosity is linked with broader immune recognition of pathogens and parasites (Zaidi et al., 2019). Relations between T and facial masculinity are also unclear. A meta-analysis found no association between the facial width-to-height ratio (FWHR) and circulating T in men (Bird et al., 2016), yet some evidence has linked T to the FWHR in peri-pubertal samples (Welker et al., 2016).

Male height has also been considered as a signal of underlying genetic quality. Despite positive assortative mating for height, women prefer men who are *relatively* taller than they are in laboratory studies, national surveys, and personal ad responses (Pawlowski and Koziel, 2002; Pawlowski, 2003; Stulp et al., 2013). Male height appears unrelated to circulating T, but rather has been linked positively to T response during exertion (Kowal et al., 2021). Height has been also linked to MHC heterozygosity (Zaidi et al., 2019). Height positively predicted men’s response to a hepatitis B vaccine up to about six feet tall, after which the trend reversed (Krams et al., 2014), whereas other research has found no links between height and immune markers in an energy-rich Western sample (Pawłowski et al., 2017). In a high pathogen threat subsistence-based sample, height for age was lower among those higher in immune markers, suggesting a potential trade-off between growth and immune function (Garcia et al., 2020) that correspond with recent evidence linking early pubertal development with less MHC heterozygosity (Arnocky et al., 2021).

The signaling properties of secondary sex characteristics have also been applied to females. Human males can benefit their reproductive fitness *via* long-term mating with healthy females, and females will benefit from outcompeting rivals for the most desirable males (Arnocky and Vaillancourt, 2017). Recent work has examined female breast symmetry, as one of our species’ most sexually dimorphic traits that have seemingly evolved to be larger than necessary for feeding young. Locke and Arnocky (2021) found that regardless of size or volume, women with symmetrical breasts were higher in salivary immunoglobulin-A (SIgA).

As demonstrated above, positive phenotypic correlations between immune markers and ornaments could be interpreted as evidence in support of parasite models (Reid et al., 2005). However, parasite models assume a causal mechanism where one is not typically tested, and most studies fail to consider specific genetic mechanisms that might underlie observed relations between immunity and physical features (Reid et al., 2005). Research would benefit from examining immune function x hormone interactions during key developmental periods in relation with downstream phenotypic development, instead of relying solely on cross-sectional assessment of these variables in adulthood. The complexity of measuring immunocompetence, in which varying systems and processes may relate differently to testosterone (Roberts et al., 2004; Hau, 2007; Nowak et al., 2018), further contributes to the challenge of interpreting the role of immune-linked traits as costly simply because they are T-dependent. Some immune markers, such as SIgA, appear to be positively related with testosterone, which could suggest that T-linked traits that correlate positively with SIgA act more as an index signal than a costly signal. The study of parasite models is further complicated by debate about directional interpretation of links between both T and immune function, and phenotypic traits and immune function, as supporting evidence, whereby negative, positive, or even null relationships could be argued as evidence of a trade-off between immunity and “costly” androgens resulting in the development or maintenance of a trait (Getty, 2002; Scott et al., 2013). Although written a decade ago, Scott et al. (2013) interpretation that “at present, there is no clear evidence of a general, cross-species link between testosterone, genetically mediated immunity, phenotypic health and trait size, from which patterns among humans can straightforwardly be inferred” hold true today (p. 581).

Collectively, these findings cast some doubt on the conclusion that well-developed secondary sexual characteristics (e.g., facial masculinity) are attractive because they evolved to signal the provisioning of direct benefits (e.g., the good-provider model) or indirect genetic benefits in the form of good genes and pathogen resistance that can be transmitted to offspring. Here the Wallacean utilitarian perspective and the importance of natural selection in governing mate choice is given prominence in adaptationist programs of research. This favoritism deviates from Darwin’s esthetic view, what Prum calls the “beauty happens hypothesis,” and alternative models of sexual selection, such as Fisherian selection, are given little attention. Particularly regarding men’s secondary sexual characteristics, there is likely value in following Prum’s (2010, 2012) LK null Model: first assuming that these traits may have incidentally evolved to be attractive because of stochastic evolutionary forces (e.g., genetic drift), and/or due to their coincidence with a third, unaccounted variable (e.g., sensory bias).

### Recommendations for Researchers Moving Forward

The LK null model itself is a quantitative genetic model with specific parameters in line with Fisherian selection (see Appendix in Prum, 2010). This is not the typical kind of empirical research and modeling undertaken by evolutionary psychologists. This raises the question of how evolutionary psychologist can apply the insights of the LK null model in their work? Part of the value of Prum’s proposal involves a theoretical shift that investigators should first not assume that mate preferences for display traits are underpinned by “extrinsic factors” such as good genes, condition-dependence, parasite avoidance, parental investment, and/or sensory bias—What Prum (2010, p. 3086) refers to as additional sources of “natural selection on mating preferences”. It involves considering that the mere existence of genetic variability in ornaments and preferences paired with assortative mating creates direct selective pressure on display traits, in addition to indirect selective pressure on the preference because of its genetic linkage to the ornament (so-called “intrinsic” forces of selection; Prum, 2010, p. 3088). Prum argues that the LK null model is more parsimonious and that we should presume that preferences for particular characteristics are the products of runaway co-evolution until there is compelling evidence favoring the operation of extrinsic factors. Consequently, the LK null model encourages scholars to raise the standard of evidence required to adhere to models of sexual selection that predominate adaptationist programs of research, such as the good genes model.

Indeed, there are ways to improve existing good genes and phenotypic condition research. For example, much of the empirical work on good genes has been centered on inconspicuous morphological characteristics (e.g., asymmetry in finger length) that are likely inconsequential to intersexual selection. It is more sensible to study heritable well-developed sexually dimorphic secondary sexual characteristic that are evidently involved in mate choice (e.g., breasts) to test for the presence of good genes and condition-dependence (Møller and Pomiankowski, 1993; Locke and Arnocky, 2021). Furthermore, characteristics that show considerable cross-cultural variability in attractiveness are unlikely to honestly communicate information about good genes, health, reproductive value, fecundity, or fertility. For instance, it is commonly believed that facial dimorphism (i.e., facial femininity and masculinity) is an honest and reliable signal of good genes and greater immunocompetence, but most of the evidence in support these ideas are based in developed and urbanized Western contexts (see Scott et al., 2013). Cross-cultural research, however, indicates substantial variability in facial dimorphism (Kleisner et al., 2021) and preferences for facial dimorphism (Scott et al., 2014) that contrast with predictions from the “parasite model.” This variability is arguably more in line with Fisherian co-evolutionary dynamics embodied within Prum’s LK null model. More cross-cultural research of a similar vein on various secondary sexual characteristics is needed.

Furthermore, when studying the links between attractive display traits, health, and immunocompetence, the focus of research should be on markers of immune function that are meaningfully tied to health outcomes, such as chronic inflammatory activity (Cunningham et al., 2022). It is also necessary to examine the collective action of multiple markers of health, rather than a small number of isolated indicators (Foo et al., 2017). For example, Mengelkoch et al. (2022) found sex-differentiated links between multiple direct *in vivo* (e.g., inflammation) and *in vitro* (e.g., growth of *Staphylococcus aureus*) markers of immune function with perceptions of facial attractiveness. Nonetheless, there seems to be limited compelling evidence to date in support of the argument that facial attractiveness is an honest and reliable signal of greater immunocompetence (Jones et al., 2021).

There is also a need for longitudinal work examining the development of phenotypic characteristics and their links with markers of good genes, health, and immune function over time during pivotal periods of development, such as late childhood and early adolescence. This kind of research is necessary to decipher the relative costs and benefits of expressing display traits and will help to clarify some of the equivocal relations between these traits with viability indicators. For instance, do markers of genetic quality predict the expression of well-developed secondary sexual characteristics? Researchers could also examine downregulation in the expression of display traits in relation to health status. For example, darker manes on male lions are preferentially desired by females perhaps as a signal of greater phenotypic condition, because darker manes might carry a cost to survival in terms of less efficient heat dissipation (West and Packer, 2002). Some indirect evidence indicates that male lions with poor nutritional status have lighter colored manes (West and Packer, 2002). Among mammals, being sick and having poor nutrition might reduce hair growth and coloration; making hair look unhealthy. Others are skeptical about the evidence in favor of this idea and note that pelage does not always correlate with nutritional status in mammals (Hill and McGraw, 2003). Like the manes of lions, men’s beards constitute a sexually dimorphic secondary sexual characteristic that women display cross-culturally variable preferences for (Dixson and Brooks, 2013). Despite some speculation (Dixson and Vasey, 2012), there is very little research supporting that beards are immunologically “costly” and that they advertise superior immune functioning.

### Criticisms of the LK Null Model

Not all evolutionary scientists, however, agree with Prum’s “beauty happens” hypothesis and the proposal that the LK null model should be the default model of sexual selection (Kempenaers, 2017; Borgia and Ball, 2018). For example, Patricelli et al. (2019) argued that Prum ignored other hypotheses for sexual displays other than Fisherian selection and stated that “Mate choice learning, as well as mutation-order divergence, sensory drive, and bias, sexual conflict, and male–male competition all provide testable nonmutually exclusive alternatives to both Fisherian and indicator models” (p. 120). Borgia and Ball (2018) also drew attention to meta-analytic work whereby little evidence was found to support genetic correlations between male sexual displays and female preferences (Greenfield et al., 2014). Although, some have pointed out that most empirical work examining trait–preference correlations have been statistically underpowered (Sharma et al., 2017). It has also been contended that because Fisher’s process has testable causal relations that it is an inappropriate null model for intersexual selection (Patricelli et al., 2019). For instance, like good genes models of sexual selection, Fisherian selection requires that: (1) a mate preference is heritable and has a genetic basis, (2) a preference is reliably linked to a specific display trait, and (3) that there is a genetic correlation between the display trait and the preference for that trait (i.e., that they are in disequilibrium). However, a null *hypothesis* is not the same as a null *model* (discussed in Kovaka, 2020). A null hypothesis is that there is no statistically significant association between observed variables (i.e., no effect). Null modeling involves comparing the most parsimonious model including a focal set of variables intended to explain some pattern in nature against a model with an additional process or mechanism (Bausman, 2018).

It is also worth mentioning that some authors have advanced alternative hypotheses that run in contrast to sexual selection. For example, Roughgarden (2012) has argued that, contrary to sexual selection, social selection offers a framework whereby mate choice functions principally to create the social conditions necessary to support offspring development. From this viewpoint, the factors implicated in nurturing and rearing offspring guide mate choice: “Social selection as presented here offers an alternative to sexual selection both as an explanation for the evolution of ornaments and as a general approach to mating behavior and parental investment” (Roughgarden, 2012, p. 2301). It is an intriguing proposal but needs to be reconciled with decades of evidence showing how humans select mates based on short-term mate value characteristics that have little to do with creating favorable conditions for offspring development (Schmitt et al., 2001).

## Conclusion

Despite cogent reviews of the various evolutionary processes that can drive sexual selection and mate preferences (e.g., Gangestad and Thornhill, 1997; Miller, 1998; Gangestad, 2001; Frederick et al., 2013), research in evolutionary psychology still appears to be guided by the assumption that traits principally become attractive because they constitute adaptations that are reliably associated with direct benefits (e.g., parental investment) and indirect genetic benefits (e.g., good genes). In doing so, the possibility that such traits may be exaptations or spandrels is discounted (Gould, 1991), as well as whether attractive features are desired because they play off pre-existing sensory biases. This approach also deviates from Darwin’s original stance articulated in *The Descent* that mate preferences can evolve for somewhat arbitrary reasons, which is captured in certain indirect benefit models of sexual selection (e.g., Fisherian runaway). But this position regarding mate choice is not given serious consideration as a competing explanation for the evolution of human mate choice. Prum (2010, 2012, 2017) advocates that the LK model should be the true null model for sexual selection and that only in the face of compelling evidence for the role of genetic and phenotypic condition should these alternative positions be favored. This may be particularly relevant for research on the attractiveness of various secondary sexual characteristics and morphological traits. The insights provided through the LK null model can help to acknowledge problematic assumptions underlying adaptationist frameworks that predominate evolutionary psychological research and to guide future work on the various models of sexual selection that collectively shape trait–preference co-evolutionary dynamics.

## Author Contributions

AD and SA wrote and edited the manuscript draft. All authors contributed to the article and approved the submitted version.

## Conflict of Interest

The authors declare that the research was conducted in the absence of any commercial or financial relationships that could be construed as a potential conflict of interest.

## Publisher’s Note

All claims expressed in this article are solely those of the authors and do not necessarily represent those of their affiliated organizations, or those of the publisher, the editors and the reviewers. Any product that may be evaluated in this article, or claim that may be made by its manufacturer, is not guaranteed or endorsed by the publisher.
